# Primary Hyperparathyroidism as a Rare Cause of Unexplained Recurrent Abdominal Pain: Case Presentation and Literature Review

**DOI:** 10.7759/cureus.19155

**Published:** 2021-10-31

**Authors:** Soliman M Bin Yahib, Bader Algarni, Abdulaziz Alghamdi, Safi Nassan

**Affiliations:** 1 Pediatric Surgery, King Abdulaziz Medical City, Jeddah, SAU; 2 Pediatric Surgery, King Saud Bin Abdulaziz University for Health Sciences College of Medicine, Jeddah, SAU

**Keywords:** abdominal pain, adolescents, pediatric, hyperparathyroidism, adenoma

## Abstract

Abdominal pain is a common symptom in surgical practice. Around 11%-45% of pediatric population present with abdominal pain. In 29%-87.5% of pediatric population diagnosed with primary hyperparathyroidism (PHPT), abdominal pain and other gastrointestinal (GI) symptoms were the presenting complaint. Hyperparathyroidism is a condition characterized by increased parathyroid hormone (PTH) secretion. It usually presents with nonspecific symptoms of fatigue, poor appetite, weight loss, abdominal pain, nausea, emesis, and bone pain.

We present a case of a 13-year-old girl who experienced a recurrent abdominal pain associated with nausea and vomiting, which was diagnosed eight months later when her laboratory investigation revealed high amylase, calcium, and PTH, which raised a suspicion of pancreatitis secondary to hyperparathyroidism. Imaging studies showed retrosternal lesion within the thymus gland, most likely a thymic parathyroid adenoma. The patient's symptoms resolved following thoracoscopic thymectomy, which was performed in another center.

To assess the relationship between GI symptoms and PHPT, we reviewed 13 articles published between 2007 and 2020 in the English literature which reported 331 cases of primary PHPT and found that GI symptoms are the fourth most common presentation in patients with PHPT. In those patients, the reported incidence of GI symptoms including abdominal pain was 18.67%. Out of the 331 cases included, only one case mimicked our case as abdominal pain was the main presenting symptom.

Unexplained recurrent abdominal pain should raise the suspicion for rare causes. Hyperparathyroidism should be included in the differential diagnosis of recurrent abdominal pain.

## Introduction

Recurrent abdominal pain is not an uncommon symptom in children and is frequently described as nonspecific [1]. However, 8-30% of pediatric patients presenting with abdominal pain can originate from organic conditions [1]. In one study by Belcher et al., gastrointestinal (GI) symptoms including abdominal pain, nausea, and constipation were the main complaints in 29%-87.5% of the pediatric population diagnosed with hyperparathyroidism [2]. Another study by Abboud et al. demonstrated that vague abdominal pain comprises 29% of symptomatic pediatric patients with hyperparathyroidism [3].

Hyperparathyroidism is a condition characterized by increased parathyroid hormone (PTH) secretion. Patients with hyperparathyroidism usually present with nonspecific symptoms of fatigue, poor appetite, weight loss, abdominal pain, nausea, emesis, and bone pain [4]. Serum calcium and serum PTH level are essential to establish the diagnosis. Technetium (99mTc) sestamibi scan is performed to locate the hyperfunctioning gland [5]. Ectopic parathyroid adenoma was located in 22% of all cases of children and usually close to the thymus [4]. Additionally, multiple endocrine neoplasia type 1 syndrome (MEN1) was identified in 23% of children with hyperparathyroidism, whereas only 2.6% had multiple endocrine neoplasia type 2 syndrome (MEN2) [6]. Surgical treatment by parathyroidectomy is required for definitive treatment [7].

## Case presentation

**Figure 1:**
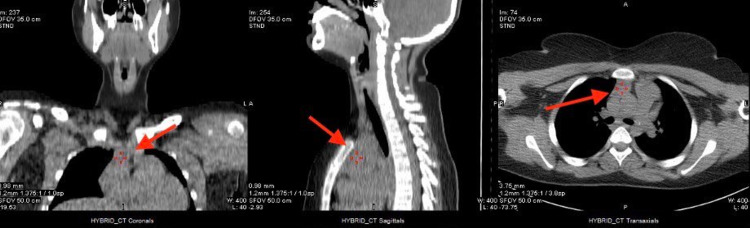
CT scan showing a hyperdense lesion in the anterior mediastinum imbedded in the thymus gland.

**Figure 2:**
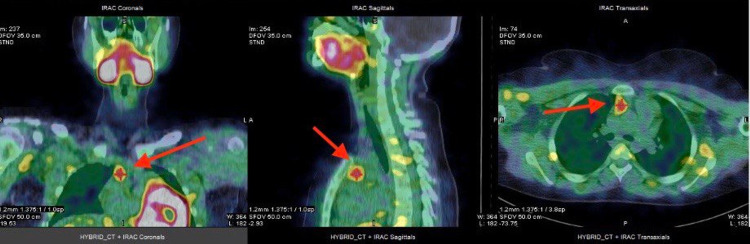
Sestamibi scan (MIBI) showing hyperactivity in the anterior mediastinum, most likely representing an ectopic parathyroid adenoma.

We report the case of a 13-year-old girl who was medically and surgically free with an eight months history of vague recurrent attacks of abdominal pain, nausea, vomiting, and migratory joint pain. During her several visits to medical centers, the physical examination was unremarkable, but at her last presentation to a higher medical center her laboratory investigation revealed elevated amylase and lipase indicating pancreatitis. At this stage, the patient was transferred to our center for further investigations. Further laboratory investigation showed high PTH (339.4 pg/mL; normal range: 22-88 pg/mL), total calcium (3.02 mmol/L; normal range: 2.2-2.7 mmol/L), adjusted calcium (2.83 mmol/L; normal range: 2.10-2.55 mmol/L), and total 25-OH vitamin D (35.2 nmol/L; optimal: >75 nmol/L). With this high PTH and calcium level, diagnosis of primary hyperparathyroidism (PHPT) was established. CT scan was performed, which showed a lesion in the anterior mediastinum, as seen in Figure 1. To localize the hyperfunctioning gland, sestamibi scan was performed, as seen in Figure 2. Genetic testing ruled out MEN1 and MEN2. The patient then underwent laparoscopic thymectomy in a different center, and the family reported that the symptoms had resolved.

## Discussion

In order to assess the relationship between GI symptoms and PHPT, we reviewed 13 articles published between 2007 and 2020 in the English literature, which reported 331 cases of primary PHPT, and found that GI symptoms are the fourth most common presentation in patients with PHPT. There were no reports in the English literature discussing PHPT as a primary cause of recurrent vague abdominal pain. Search terms included “primary hyperparathyroidism”, “children”, “pediatrics”, “adolescents”, “adenoma”, “ectopic”, and “hyperplasia”. Ages range from 0 to 19 years. Retrospective review studies, literature reviews, and case reports were included. Parathyroid adenoma, symptoms, and management were the main focus, whereas other parathyroid pathologies such as hyperplasia and carcinoma were excluded. We ended up yielding a total of 35 articles. After excluding the articles that did not meet the criteria, we used 13 articles, as seen in Table 1, with a number of 331 patients. Eleven of these articles reported the site of adenoma. In those patient, the reported incidence of GI symptoms including abdominal pain were 18.67%. Table 1 demonstrates the studies, date of publication, number of patients, age, site of adenoma, and the main presenting symptom. Out of the 331 cases included in the table, only one case mimicked our case as abdominal pain was the main presenting symptom.

**Table 1:** Articles used in the literature review ^a^One patient was 22 years old at the time of surgery but had documented symptoms for 12 years. ^b^The toddler did not have adenoma but hyperparathyroidism due to Williams-Beuren syndrome. GI, gastrointestinal

Title	Year	Study type	Number of patients	Age range (years)	Site	Presenting symptoms
Characterization of hyperparathyroidism in youth and adolescents: a literature review [2]	2013	Literature review	230	4-22^a^	-	Renal (30%), bone disease (23,91%), fatigue and lethargy (18.2%), gastrointestinal (16.52%), neurological (7.8%), polydipsia (2.17%)
Comparative characteristics of primary hyperparathyroidism in pediatric and young adult patients [6]	2016	Cohort	39	8.8-18.6	-	Fatigue and depression (53.8%), incidental (46%), renal (43.59%), neurological (30.7%), muscle pain (23%), GI (19.5%), asymptomatic (17.9%)
Primary hyperparathyroidism in children and adolescents [8]	2010	Retrospective	18	13-20	2 right superior, 7 right inferior, 1 left superior, 8 left inferior	Myopathy (50%), bone (88.9%), gastrointestinal (5.5%), renal (38.9%)
Surgical treatment of children with hyperparathyroidism: single center experience [9]	2014	Retrospective case series	29	0-16	-	Gastrointestinal (48.28%), bone (20.7%), neurological (20.7%), lethargy (10.34%), renal (6.9%)
Solitary parathyroid adenoma a rare cause of hyperparathyroidism in children [10]	2007	Case series	4	14-17	4 Left inferior	Gastrointestinal (75%), neurological (50%), renal (50%), bone disease (25%)
An occult ectopic parathyroid adenoma in a pediatric patient: a case report [4]	2017	Case report	1	13	Ectopic "thymic"	Inability to concentrate and worsening generalized and muscle fatigue
Ectopic thymic parathyroid adenoma and vitamin D deficiency rickets: a 5-year-follow-up case report and review of literature [11]	2008	Case report	1	14	Ectopic “thymic”	Bilateral foot pain and difficulty walking
Minimally invasive resection of mediastinal parathyroid adenoma using SPECT/CT and intact PTH monitoring [12]	2011	Case report	1	10	Ectopic “mediastinal”	2-week history of abdominal pain and acute pancreatitis
Undescended retropharyngeal parathyroid adenoma with adjacent thymic tissue in a 13-year-old boy with primary hyperparathyroidism [13]	2019	Case report	1	13	Ectopic “Retropharyngeal”	Presented to the emergency department with a 3-day history of fever and sore throat with associated difficulty swallowing
Parathyroid adenoma presenting as genu valgum in a child: a rare case report [14]	2019	Case report	1	12	Right inferior	Presented with pain in bilateral knee joints and gait abnormality for one year, physical examination revealed genu valgum
Two different causes of pediatric hypercalcemia [15]	2018	Case report	2	7 months, 16	Left superior^b^	Failure to thrive, vomiting and psychomotor retardation; abdominal pain and renal colic due to hypercalcemia-induced urolithiasis
Incidental hypercalcemia caused by primary hyperparathyroidism with rapid progression to renal complications in a child [16]	2018	Case report	1	8 years and 9 months	Left subclavicular	Incidental
Parathyroid adenoma [17]	2008	Case report	1	10	Dorsolateral to left common carotid artery	Fatigue

Table 2 illustrates that GI symptoms including abdominal pain were the fourth most common presenting symptom of hyperparathyroidism in pediatrics and adolescent. Patients also presented with other symptoms, as shown in Table 2.

**Table 2:** Frequency of presenting symptoms GI, gastrointestinal

Main symptom	%	including
Renal	27.41	Nephrolithiasis polyuria
Bone disease	23.49	Bone pain bone deformity fractures
Lethargy and fatigue	20.48	-
GI	18.67	Abdominal pain, weight loss, anorexia, jaundice
Neurological	17.46	Depression memory loss inability to concentrate
Incidental	5.44	-
Asymptomatic	3.31	-

The diversity of nonspecific symptoms such as inability to concentrate, headaches, bone pain, deformity, fractures, fatigue, growth retardation, and abdominal pain makes the diagnosis challenging to physicians [2]. Since these symptoms are common in pediatric and adolescent age groups, the diagnosis is considered challenging. Furthermore, 91% of symptomatic pediatrics and adolescents have had severe complications such as nephrolithiasis, nephrocalcinosis, pancreatitis, bone and joint deformities, and fractures [2]. Since unexplained recurrent abdominal pain could be the only presenting symptom, hyperparathyroidism should be included in the differential diagnosis.

## Conclusions

Unexplained recurrent abdominal pain should raise the suspicion for rare causes. Hyperparathyroidism should be included in the differential diagnosis of recurrent abdominal pain. Diagnosis of PHPT is almost delayed in all children with atypical symptoms. For that reason, we presented this case to draw attention to the diagnosis of PHPT with recurrent abdominal pain as the main symptom in order to prevent further complications.
